# Pan-genome analysis of the *Enterobacter hormaechei* complex highlights its genomic flexibility and pertinence as a multidrug resistant pathogen

**DOI:** 10.1186/s12864-025-11590-1

**Published:** 2025-04-26

**Authors:** Pieter De Maayer, Teigra Green, Sara Jordan, Theo H. M. Smits, Teresa A. Coutinho

**Affiliations:** 1 School of Molecular and Cell Biology, University of the Witwatersrand, Johannesburg, 2000 South Africa; 2 Environmental Genomics and Systems Biology Research Group, Institute for Environment and Natural Resources, Zürich University for Applied Sciences (ZHAW), Wädenswil, Switzerland; 3 Department of Microbiology and Plant Pathology, Centre for Microbial Ecology and Genomics/Forestry and Agricultural Biotechnology Institute (FABI), University of Pretoria, Pretoria, 0002 South Africa

**Keywords:** *Enterobacter hormaechei*, Pan-genome, Comparative genomics, Multidrug resistance, Nosocomial pathogen

## Abstract

**Background:**

*Enterobacter hormaechei* is of increasing concern as both an opportunistic and nosocomial pathogen, exacerbated by its evolving multidrug resistance. However, its taxonomy remains contentious, and little is known about its pathogenesis and the broader context of its resistome. In this study, a comprehensive comparative genomic analysis was undertaken to address these issues.

**Results:**

Phylogenomic analysis revealed that *E. hormaechei* represents a complex, comprising three predicted species, *E. hormaechei, E. hoffmannii* and *E. xiangfangensis*, with the latter putatively comprising three distinct subspecies, namely *oharae, steigerwaltii* and *xiangfangensis*. The species and subspecies all display open and distinct pan-genomes, with diversification driven by an array of mobile genetic elements including numerous plasmid replicons and prophages, integrative conjugative elements (ICE) and transposable elements. These elements have given rise to a broad, relatively conserved set of pathogenicity determinants, but also a variable set of secretion systems. The *E. hormaechei* complex displays a highly mutable resistome, with most taxa being multidrug resistant.

**Conclusions:**

This study addressed key issues pertaining to the taxonomy of the *E. hormaechei* complex, which may contribute towards more accurate identification of strains belonging to this species complex in the clinical setting. The pathogenicity determinants identified in this study could serve as a basis for a deeper understanding of *E. hormaechei* complex pathogenesis and virulence. The extensive nature of multidrug resistance among *E. hormaechei* complex strains highlights the need for responsible antibiotic stewardship to ensure effective treatment of these emerging pathogens.

**Supplementary Information:**

The online version contains supplementary material available at 10.1186/s12864-025-11590-1.

## Background

*Enterobacter hormaechei* is a Gram-negative, facultatively anaerobic, rod-shaped bacterium belonging to the family *Enterobacteriaceae* [1]. This species is frequently isolated from environmental sources such as soil, water, food and the hospital setting, and forms associations with plant, insect and animal hosts [2]. However, it is the pathogenic association of this species with humans that has received the most attention. It has been recognised as a significant opportunistic pathogen, with clinical manifestations ranging from bacteraemia, pneumonia, surgical site infections to urinary tract infections [2, 3]. Particularly in the healthcare setting, this pathogen poses a significant threat to human health, with *E. hormaechei* being the most frequently isolated species among the genus *Enterobacter* [3, 4].

One of the most concerning aspects of *E. hormaechei* is its broad-scale antibiotic resistance, with strains often exhibiting multidrug resistance phenotypes [3]. This pathogen harbours multiple genes conferring resistance to antibiotics such as amikacin, ciprofloxacin, cefoxitin, colistin, gentamicin, tigecycline, as well as carbapenems [2]. The ability of this species to acquire and spread resistance genes through horizontal gene transfer exacerbates the issue, making it a formidable adversary in the clinical setting [2, 3]. As such, *E. hormaechei* is included in the ESKAPE group (*Enterococcus faecium*, *Staphylococcus aureus*, *Klebsiella pneumoniae*, *Acinetobacter baumannii*, *Pseudomonas aeruginosa* and *Enterobacter* spp.), a collection of pathogens responsible for a significant proportion of hospital-acquired infections and renowned for their ability to evade the effects of antibiotics [5]. The inclusion of *E. hormaechei* in this group underscores its importance as a target for infection control and antibiotic stewardship efforts.

Despite its clinical significance, several aspects of the biology of *E. hormaechei* remain poorly understood. A primary issue is the complexity of its taxonomy. This species currently comprises five distinct subspecies, namely, *hormaechei, hoffmannii, oharae, steigerwaltii* and *xiangfangensis* [6]. Standard phenotypic tests often fail to distinguish between the subspecies and other members of the genus, necessitating the use of advanced molecular techniques such as whole genome sequencing for precise identification [1, 7]. This has led to the suggestion that subspecies *hoffmannii* and *xiangfangensis* should be elevated to species status as *E. hoffmannii* and *E. xiangfangensis*, respectively [1, 7]. Accurate classification and identification is key for effective infection control and treatment strategies, particularly in light of the distinct clinical manifestations of *E. hormaechei* and its subspecies, with *E. hormaechei* subsp. *oharae* being more prevalent in blood cultures, whereas *E. hormaechei* subsp. *steigerwaltii* was overrepresented in skin and burn injury swabs [4]. A second issue is that, while its resistance to antibiotics has received extensive attention, research has tended to focus on specific clinical *E. hormaechei* isolates, which in the context of the taxonomic complexity, makes effective antibiotic treatment strategies difficult [2]. Furthermore, in contrast to antibiotic resistance, limited research has been done on the pathogenicity and virulence determinants of this bacterial species, adding to the difficulty of effective treatment of *E. hormaechei* infections.

To address these issues, in this study we performed comparative genomic analyses on a comprehensive set of *E. hormaechei* isolates. Through phylogenomic analyses, a better resolution of its classification was developed. By means of pan-genome analysis, the open pan-genome of *E. hormaechei* was elucidated and we identified several drivers of the genotypic diversification of the complex species. Furthermore, the comparative genomic analyses provided prime insights into the global resistome and pathogenome of *E. hormaechei.*

## Results

### The *E. hormaechei* complex comprises three distinct species and three subspecies

**Fig. 1:**
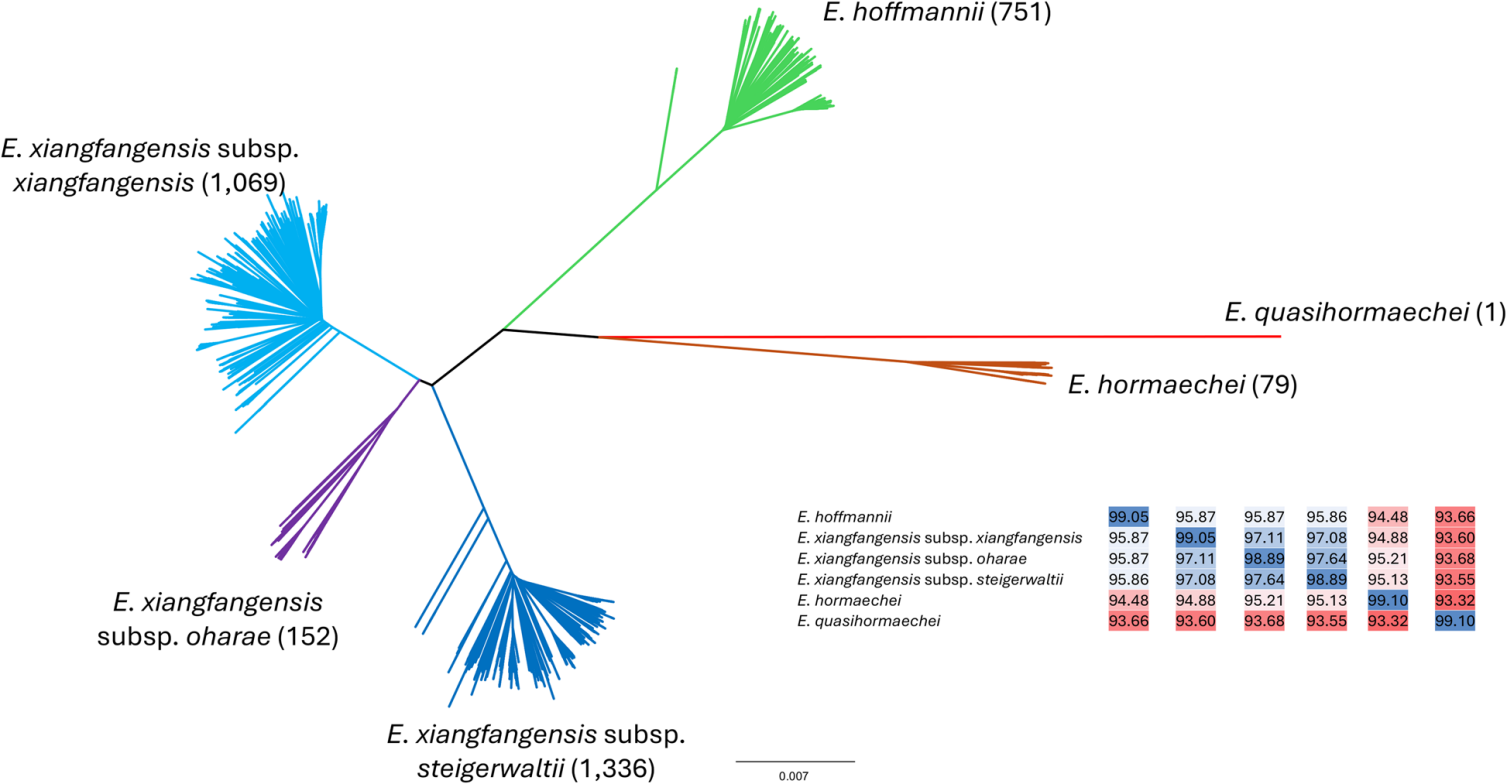
NJ phylogeny of ANI values of 3,387 *E. hormaechei* strains. ANI values were calculated using FastANI [10] and converted to distance values. The phylogeny was constructed using the *neighbor.exe* function in Phylip v. 3.698 [11]. The genome of the type strain *E. quasihormaechei* WCHEQ120003^T^ was used as outgroup. The inset shows the average ANI values within and between species/subspecies. A colour gradient shows those values above the species-level cut-off value of 95% in blue and those values below the species boundary in red

The genomes of 3,387 *E. hormaechei* strains (Supplementary Material 1 Table S1) were obtained from the NCBI assembly database [8]. The majority (87.7%) were derived from humans and medical settings, indicative of the clinical relevance of the *E. hormaechei* complex [7, 9], while 6.1% of the strains were isolated from animals and 6.2% from environmental sources, including 41 strains isolated from plant sources. The genomes were clustered on the basis of their pair-wise average nucleotide identity (ANI) values, converted to distance values and used to construct a distance tree. The resultant tree showed the clear delineation of the *E. hormaechei* strains in five distinct clades alongside the *E. quasihormaechei* WCHEQ120003^T^ outgroup (Fig. 1).

**Fig. 2:**
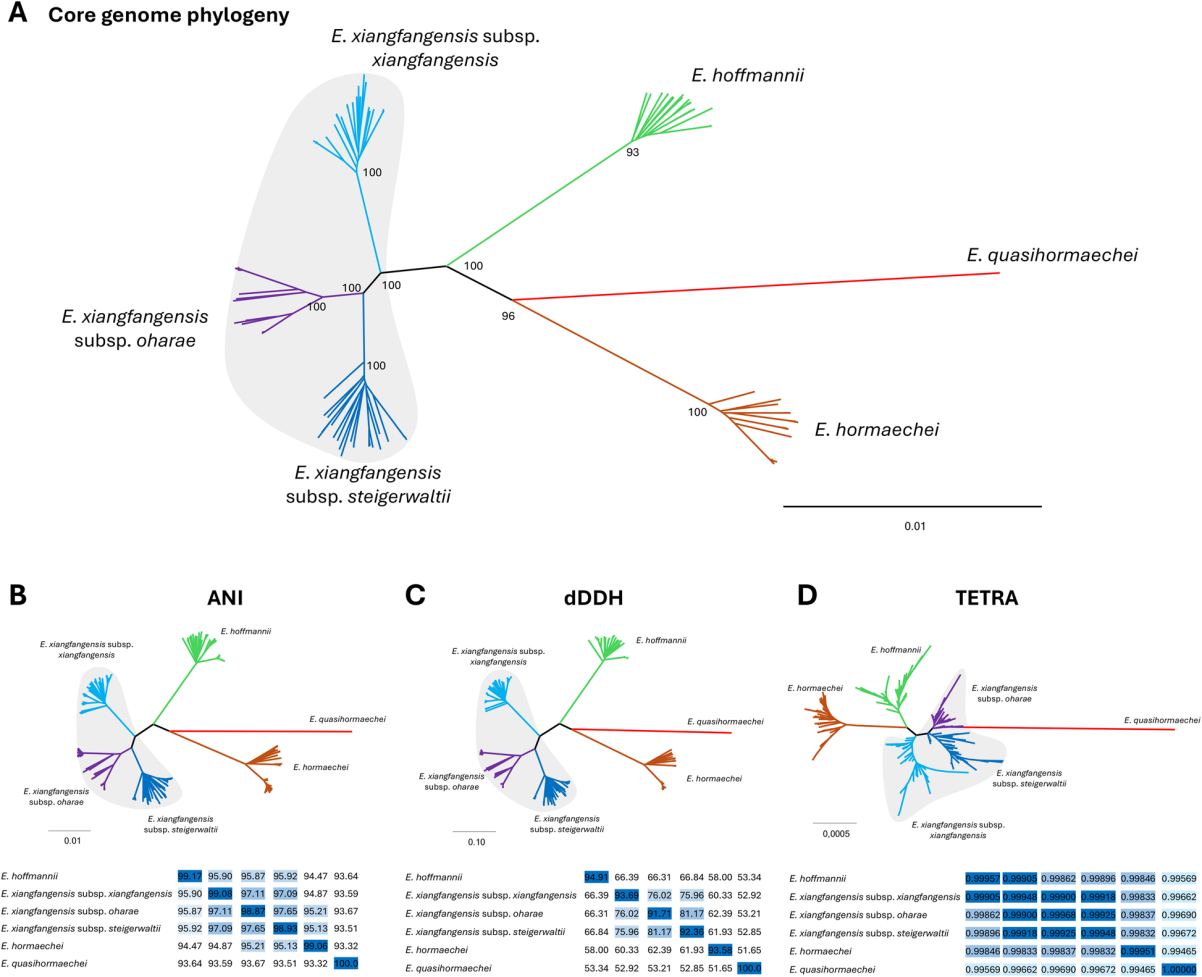
Phylogenomic analyses of 250 representative taxa of the *E. hormaechei* complex. **A** Core genome ML phylogeny constructed on the basis of 3,308 SCOs. The curated alignment comprised 980,799 amino acid positions, with 56,435 parsimony informative sites and 29,084 singleton sites. The tree was constructed using IQ-Tree v. 2.3.6 [13] with the optimal evolutionary model JTT + F + I + G4 and ultra-fast bootstrap support [14] with 1,000 replicates (percentage values are shown). **B** NJ tree of ANI distance values calculated for the 250 representative *E. hormaechei* taxa. **C** NJ tree on the basis of dDDH [15] distance values (**D**) NJ tree on the basis of TETRA [16] distance values. The genome of the type strain *E. quasihormaechei* WCHEQ120003^T^ was used as outgroup in all trees. The insets show the average intra- and inter-(sub)specific ANI (B), dDDH (**C**) and TETRA (**D**) values, respectively. Values above the respective specific boundary cut-off values of 95–96% (ANI), 70% (dDDH) and 0.99 (TETRA) are shaded in blue according to scale of magnitude

Phylogenomic analyses were conducted using 50 representatives from each clade, including the type strains of each recognised *E. hormaechei* subspecies and *E. quasihormaechei* WCHEQ120003^T^ as the outgroup (Supplementary Material 1 Table S2). A core genome phylogeny on the basis of 3,308 conserved single-copy orthologous proteins (SCOs) similarly showed delineation of the taxa into five clades, representative of the five currently recognised *E. hormaechei* subspecies **(**Fig. 2A). ANI, digital DNA-DNA hybridisation (dDDH) and tetranucleotide signature frequency correlation coefficient (TETRA) values were calculated (Supplementary Material 1 Tables S3-S5) and used to construct distance trees, with the ANI and dDDH trees displaying similar topologies to the core genome phylogeny (Fig. 2B-D). In the TETRA distance tree, the strains again clustered in five distinct clades, but with distinct branching patterns, including the incorporation of the outgroup taxon *E. quasihormaechei* WCHEQ120003^T^ within the *E. xiangfangensis* subsp. *oharae* clade and likely the effect of long-branch attraction (Fig. 2D). Tetranucleotide usage is known to be influenced by local variations in genome composition and horizontal gene transfer events [12].

ANI values of 95–96%, dDDH values of 70% and TETRA values of 0.99 have been validated to approximate the wet-lab DNA-DNA hybridisation value of 70% considered the boundary for species circumscription [15, 16]. ANI and dDDH values for strains from the clade incorporating the type strain of *E. hormaechei* subsp. *hormaechei* (ATCC 49162^T^) are below 95% and 70% when compared to strains in the other four clades (94.91% and 60.66% average, respectively), suggesting that they represent a distinct species from the other four clades. By contrast, interclade ANI and dDDH values for strains in the *E. oharae, steigerwaltii* and *xiangfangensis* clades are consistently above 96% and 70% (97.95% and 83.01% average, respectively), indicating that they belong to a single species, in congruence with a previous comprehensive comparative genomic analysis of the genus *Enterobacter* [7]. *Enterobacter xiangfangensis* was originally validly published as a distinct species [17] and should thus revert to its prior nomenclature [1]. A dDDH value of 79–80% was proposed as the boundary for the delineation of subspecies [18]. Intra-clade dDDH values between the *oharae, steigerwaltii* and *xiangfangensis* clades range between 91.71 and 93.69%, while the interclade values range between 75.96 and 81.17%, suggesting that they represent three distinct subspecies of *E. xiangfangensis*.

The fifth clade, incorporating the *E. hormaechei* subsp. *hoffmannii* type strain (DSM 14563^T^), shares borderline ANI values between 95.87 – 95.92% with strains in the clades representing *E. xiangfangensis* subsp. *oharae, steigerwaltii* and *xiangfangensis,* suggesting strains in this clade may be conspecific with the latter taxa. However, dDDH values (average 66.31 – 66.84% with the subspecies *oharae, steigerwaltii* and *xiangfangensis* strains) are below the 70% species threshold, as previously observed [7] and the distinct clustering of the *E. hormaechei* subsp. *hoffmannii* strains in all phylogenies present a case for the consideration of *E. hoffmannii* as a distinct species [1, 7]. Further phenotypic and genotypic analyses will need to be undertaken to delineate and describe the *E. hormaechei* complex species and subspecies. However, for the purpose of our comparative genomic analyses, the clusters *E. hormaechei*, *E. hoffmannii* and *E. xiangfangensis* were considered as three distinct species, with the latter comprising three clades representing *E. xiangfangensis* subsp. *oharae,* subsp. *steigerwaltii* and subsp. *xiangfangensis*.

The genomes of the three *Enterobacter* species are, on average, similar in terms of genome size, number of proteins they encode and G + C content (Table 1). However, the genomes display extensive variability among the compared taxa, ranging by up to 1.23 Mb in size (4.42—5.65 Mb) and 1.5% in G + C content (54.39 – 55.89%). Comparison of the three *E. xiangfangensis* subspecies indicated that the genomes of *E. xiangfangensis* subsp. *oharae* are on average ~ 327 kb smaller, with a G + C content 0.44% higher than those of the other subspecies. Even within individual species/subspecies, genomes vary in size, G + C content and number of proteins they encode. For example, the genomes of the *E. xiangfangensis* subsp. *steigerwaltii* strains range in size by 1.19 Mb, code for between 4,188 and 5,366 proteins and range in G + C content by up to 1.27%. Together, these metrics demonstrate that *E. hoffmannii, E. hormaechei* and *E. xiangfangensis* exhibit extensive genome flexibility.

**Table 1:** Overall genome metrics of the analysed representative *E. hormaechei* complex species and subspecies. The average and range genome sizes, number of proteins encoded on the genome and G + C contents (%) are shown

Species/subspecies	Genome size (Mb)	# Proteins	G + C content (%)
*E. hoffmannii* (50)	5.04 (4.55 – 5.56)	4,688 (4,136 – 5,316)	54.79 (54.39 – 55.41)
*E. hormaechei* (50)	4.84 (4.55 – 5.19)	4,511 (4,208 – 4,907)	55.02 (54.41 – 55.47)
*E. xiangfangensis* (150)	5.01 (4.42 – 5.65)	4,665 (4,046 – 5,366)	55.15 (54.45 – 55.89)
*E. xiangfangensis* subsp. *oharae* (50)	4.79 (4.42 – 5.22)	4,440 (4,046 – 4,895)	55.45 (54.86 – 55.89)
*E. xiangfangensis* subsp. *steigerwaltii* (50)	5.18 (4.46 – 5.65)	4,836 (4,188 – 5,366)	55.06 (54.45 – 55.72)
*E. xiangfangensis* subsp. *xiangfangensis* (50)	5.05 (4.66 – 5.55)	4,718 (4289 – 5,288)	54.95 (54.47 – 55.47)
*E. quasihormaechei* (1)	4.56	4180	55.90

### The *E. hormaechei* complex species display open pan-genomes

**Fig. 3:**
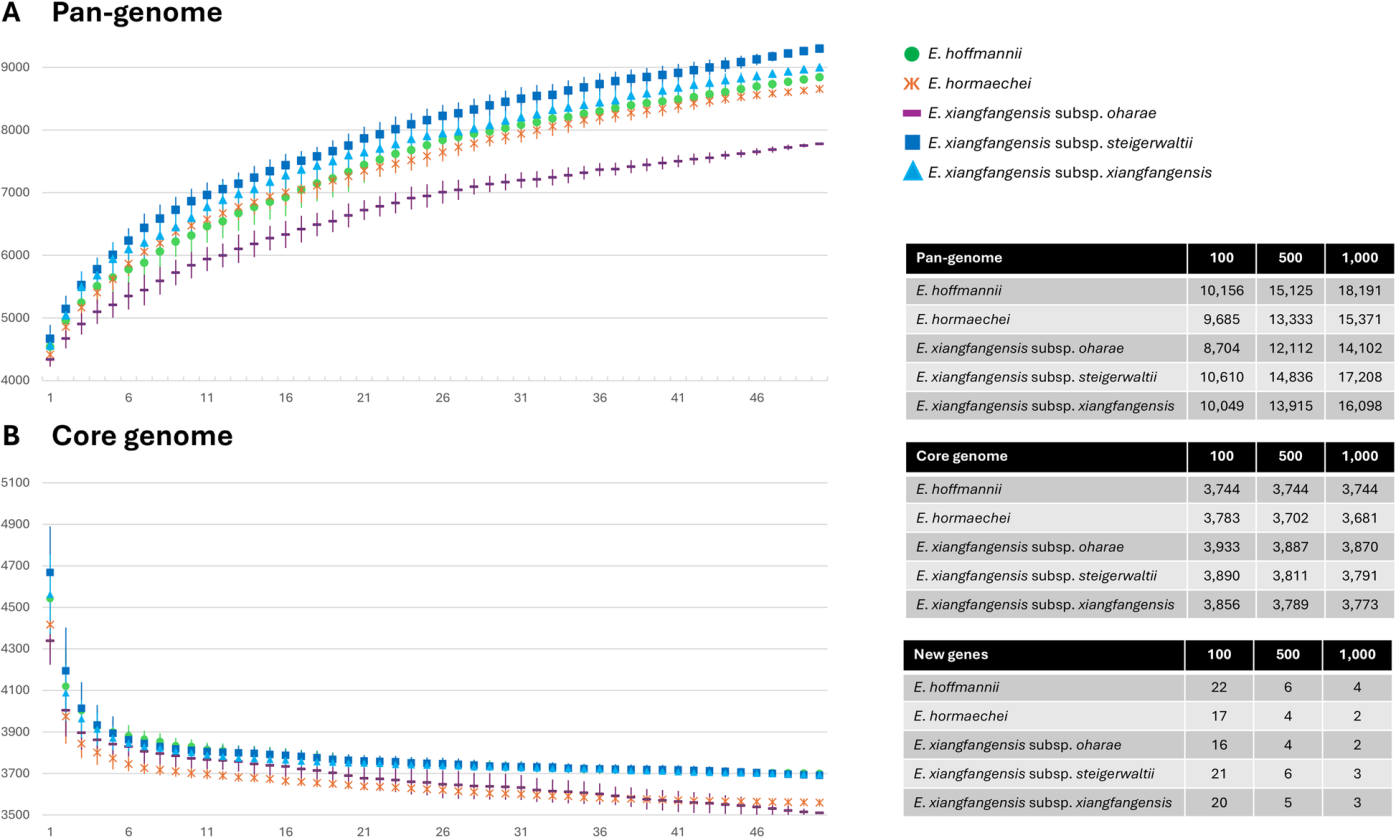
Pan-genome graphs and extrapolations for the *E. hormaechei* complex species and subspecies. **A** Pan-genome graph across the 50 representative taxa for each species/subspecies. **B** Core genome graph across the 50 representative taxa for each species/subspecies. The insets show the extrapolated pan-genome, core genome and new genes added when comparing 100, 500 and 1,000 genomes of each species/subspecies. This was calculated using Heap’s Law [19] using the curve fitting functions in PanGP [20]

To gain further insight into the genome flexibility of the *E. hormaechei* complex, protein orthology was predicted and used to construct pan- and core genome development plots for each species/subspecies. All *Enterobacter* species and subspecies displayed open pan-genomes, with the largest pan-genomes observed for *E. xiangfangensis* subsp. *xiangfangensis* (9,001 orthogroups) and *E. xiangfangensis* subsp. *steigerwaltii* (9,299 orthogroups) across 50 compared taxa (Fig. 3). By contrast, the *E. xiangfangensis* subsp. *oharae* pan-genome comprised on average 969 and 1,370 orthogroups less than the other species and *E. xiangfangensis* subspecies, respectively, demonstrating correlation with the smaller genome sizes and protein complements of *E. xiangfangensis* subsp. *oharae* strains. Notably, *E. xiangfangensis* subsp. *oharae* also displayed the smallest core genome among all compared species/subspecies, indicating that its pan-genome may be affected by genome reduction in core genomic elements, while maintaining a sizable accessory genome driving the diversification of individual strains of this subspecies. Extrapolation of the pan-genome using Heap’s Law [19] predicts that the pan-genome of *E. xiangfangensis* subsp. *xiangfangensis* and subsp. *steigerwaltii* will reach 16,098 and 17,208 orthogroups, respectively if 1,000 genomes of each would be included in the comparison, while the pan-genome of *E. xiangfangensis* subsp. *oharae* would only comprise 14,102. Notably, on the basis of the pan-genome curves, *E. hoffmannii* is predicted to have the largest pan-genome, comprising 18,191 orthogroups for 1,000 genomes. While the addition of the 1,000th genome of *E. xiangfangensis* subsp. *oharae* and *E. hormaechei* is predicted to add two novel genes to the pan-genome, and three genes in the case of *E. xiangfangensis* subsp. *xiangfangensis* and *steigerwaltii*, the 1,000th genome of *E. hoffmannii* would add four genes to the overall pan-genome of the latter species, suggestive of versatile accessory genomic elements in the *E. hormaechei* complex.

### The *Enterobacter hormaechei* complex species and *E. xiangfangensis* subspecies display extensive proteome and functional diversification

**Fig. 4:**
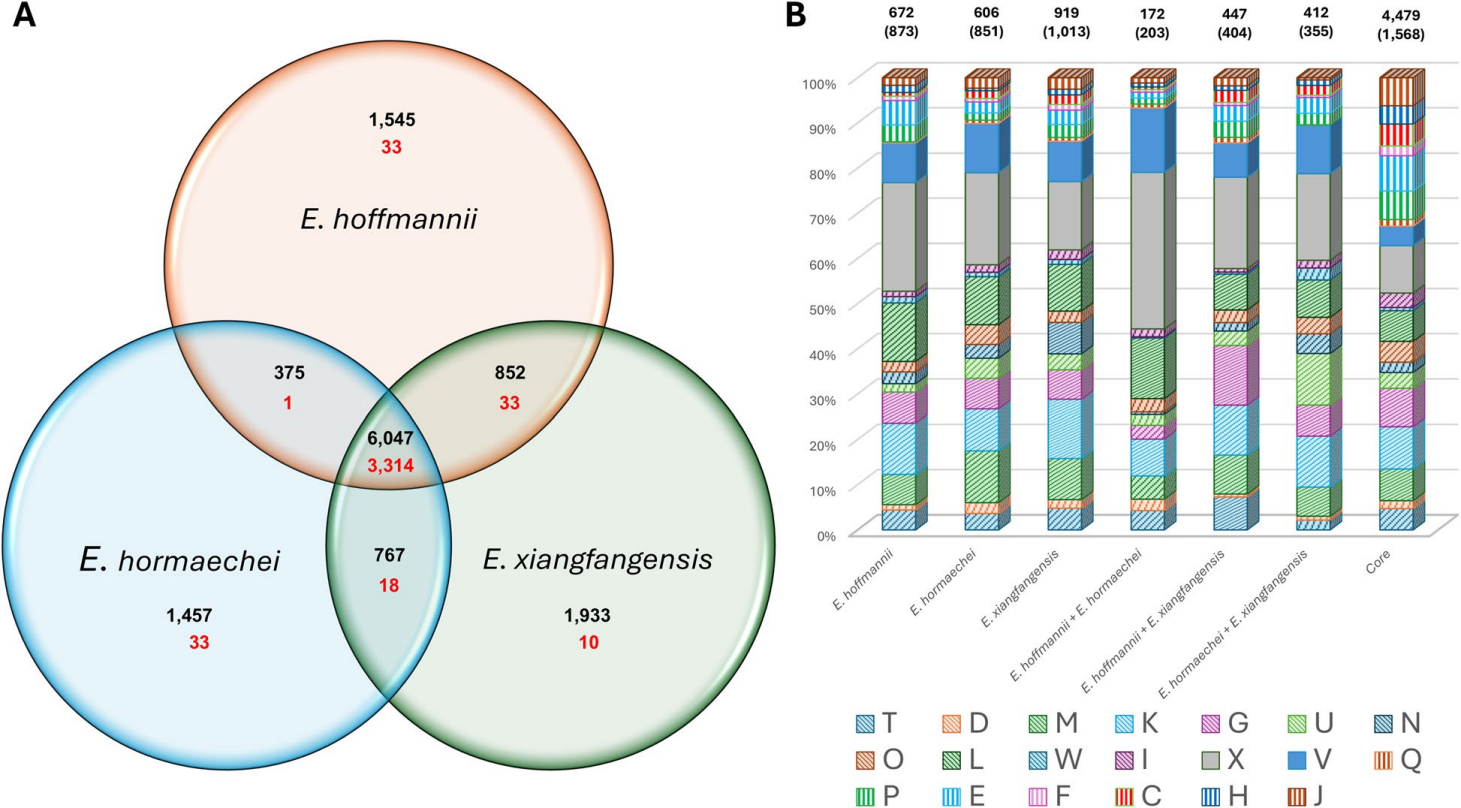
Functional analysis of the *E. hormaechei* complex pan-genome fractions. **A** Venn diagram depicting the orthogroups core to all three species, shared by two of the species and unique to a single species. Numbers in red denote those proteins conserved among all compared taxa in a pan-genome fraction. **B** Bar chart showing proportions of proteins per COG category. Bars shaded in full are those showing > two-fold representation in the accessory pan-genome fractions compared to the core fractions and bars shaded with downward hashes depict those COG functions with > two-fold representation in the core pan-genome fraction compared to the accessory fractions. The letters in the legend represent the codes for each functional category as per the COG database [21]: C: Energy production and conversion, D: Cell cycle control, cell division, chromosome partitioning, E: Amino acid transport and metabolism, F: Nucleotide transport and metabolism, G: Carbohydrate transport and metabolism, H: Coenzyme transport and metabolism, I: Lipid transport and metabolism, J: Translation, ribosomal structure and biogenesis, K: Transcription, L: Replication, recombination and repair, M: Cell wall/membrane/envelope biogenesis, N: Cell motility, O: Posttranslational modification, protein turnover, chaperones, P: Inorganic ion transport and metabolism, Q: Secondary metabolite biosynthesis, transport and catabolism, T: Signal transduction mechanisms, U: Intracellular trafficking, secretion and vesicular transport, V: Defense mechanisms, W: Extracellular structures, X: Mobilome: prophages, transposons

The combined pan-genome of *E. hoffmannii* (50 genomes), *E. hormaechei* (50 genomes) and *E. xiangfangensis* (50 randomly selected genomes among the 50 subsp. *oharae,* 50 subsp. *steigerwaltii* and 50 subsp. *xiangfangensis* genomes) comprises 12,976 orthogroups, with 46.6% of these represented in at least one taxon per species (cloud core) and only 25.5% conserved in all compared taxa (strict core) (Fig. 4A). The extensive accessory genome can largely be attributed to the orthogroups unique to the individual species, contributing between 17.5% (*E. hormaechei*) and 20.1% (*E. xiangfangensis*) of the pan-genome of each individual species. Noteworthy is the low number of species-specific orthogroups conserved among the 150 compared taxa, with on average only 1.6% of the orthogroups core to all 50 compared taxa per species, indicative of extensive proteome variability between individual strains in each species. The highest number of orthogroups shared between two species (but absent from the third) were observed for the *E. hoffmannii – E. xiangfangensis* and *E. hormaechei – E. xiangfangensis* combinations, while less than half as many proteins are shared by *E. hoffmanni* and *E. hormaechei* taxa only (Fig. 4A). Again, a restricted number of these are core to all taxa (2.3% average) in each pair combination, suggesting horizontal exchange of genes between individual strains post-speciation.

The proteomes of each pan-genome element were classified according to their Clusters of Orthologous Genes (COG) functions (Fig. 4B) [21]. The core elements showed a greater than two-fold representation of most of the metabolic functions (C: Energy production and conversion; E: Amino acid transport and metabolism; F: Nucleotide transport and metabolism; H: Coenzyme transport and metabolism; I: Lipid transport and metabolism; P: Inorganic ion transport and metabolism and Q: Secondary metabolites biosynthesis, transport and catabolism) and Translation, ribosomal structure and biogenesis (COG category J) compared to the accessory pan-genome elements. By contrast, the accessory pan-genome elements showed a greater than two-fold representation of proteins involved in Defense mechanisms (COG category V) and the Mobilome: phages, transposons and plasmids (COG category X) compared to the core pan-genome elements. Among the species-specific defense mechanisms were a number of distinct antiphage defence systems, as well as restriction endonucleases and proteins involved in antibiotic resistance.

**Fig. 5:**
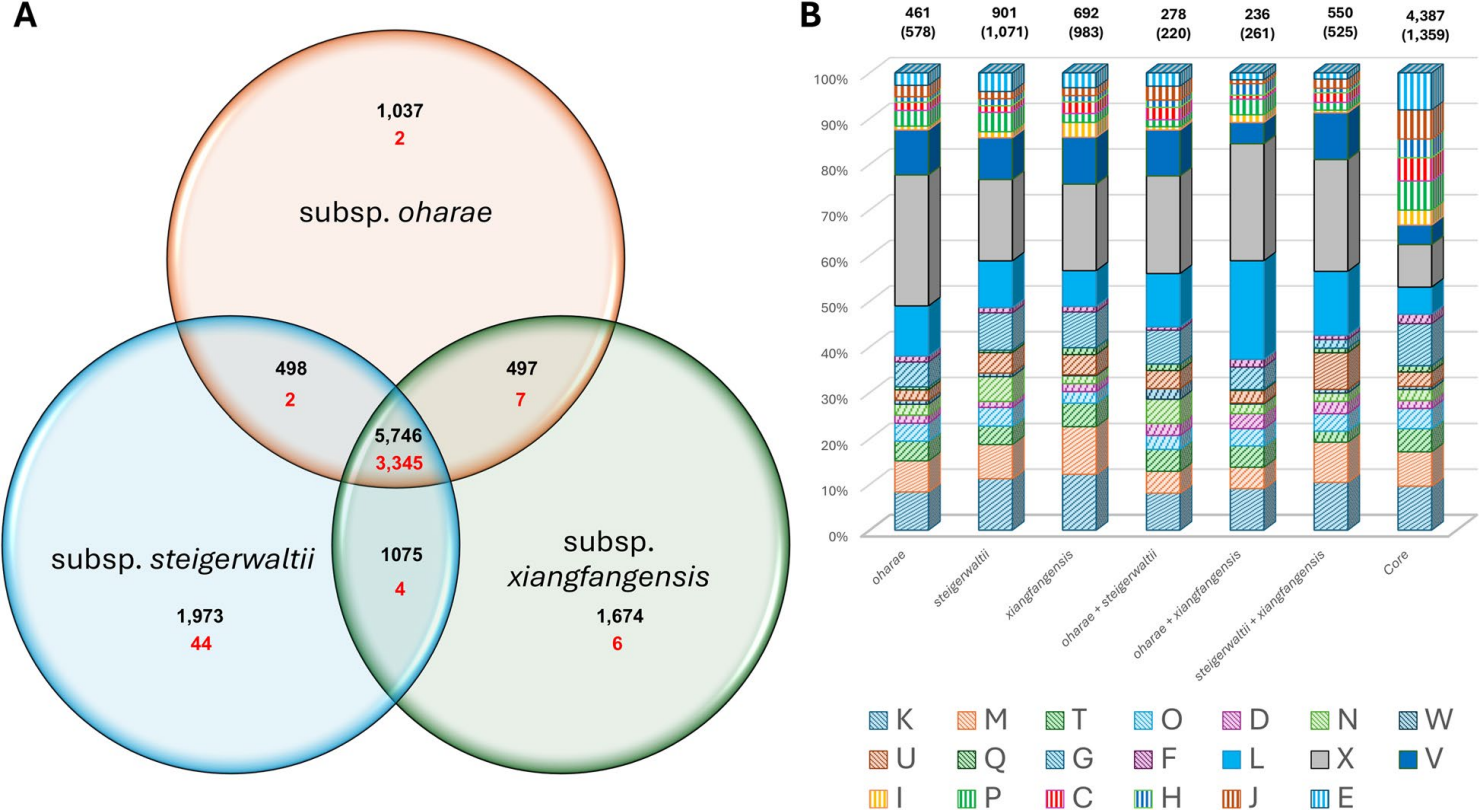
Functional analysis of the *E. xiangfangensis* subspecies pan-genome fractions. **A** Venn diagram depicting the orthogroups core to all three *E. xiangfangensis* subspecies, shared by two of the subspecies and unique to a single subspecies. Numbers in red denote those proteins conserved among all compared taxa in a pan-genome fraction. **B** Bar chart showing proportions of proteins per COG category. Bars shaded in full are those showing > two-fold representation in the accessory pan-genome fractions compared to the core fractions and bars shaded with downward hashes depict those COG functions with > two-fold representation in the core pan-genome fraction compared to the accessory fractions. The letters in the legend represent the codes for each functional category as per the COG database [21]: C: Energy production and conversion, D: Cell cycle control, cell division, chromosome partitioning, E: Amino acid transport and metabolism, F: Nucleotide transport and metabolism, G: Carbohydrate transport and metabolism, H: Coenzyme transport and metabolism, I: Lipid transport and metabolism, J: Translation, ribosomal structure and biogenesis, K: Transcription, L: Replication, recombination and repair, M: Cell wall/membrane/envelope biogenesis, N: Cell motility, O: Posttranslational modification, protein turnover, chaperones, P: Inorganic ion transport and metabolism, Q: Secondary metabolite biosynthesis, transport and catabolism, T: Signal transduction mechanisms, U: Intracellular trafficking, secretion and vesicular transport, V: Defense mechanisms, W: Extracellular structures, X: Mobilome: prophages, transposons

Analysis of the pan-genome of the three *E. xiangfangensis* subspecies showed similar trends, in that the core genome contributed 45.97% of the pan-genome (12,500 orthogroups), with only 26.76% of orthogroups core to all 150 taxa (Fig. 5A), highlighting extensive genomic variability at the subspecies level for this species. Subspecies-specific orthogroups contributed between 13.33% (subsp. *oharae*) and 21.23% (subsp. *steigerwaltii*) of the subspecies pan-genomes. The pan-genome of the former subspecies is the smallest (7,778 orthogroups), correlating to the smaller genome sizes of *E. xiangfangensis* subsp. *oharae* strains, while that of *E. xiangfangensis* subsp. *steigerwaltii* is the largest, comprising 9,292 orthogroups. Again, only a small proportion (0.89% average) of subspecies-specific proteins are conserved among all 50 compared taxa. A similar number of proteins is shared by the subspecies pairs *oharae – steigerwaltii* and *oharae – xiangfangensis* (absent from the third subspecies), while more than double as many proteins are shared by the subspecies *steigerwaltii – xiangfangensis* but are absent from *E. xiangfangensis* subsp. *oharae* strains.

As with the inter-species comparison, more than two-fold relative proportions of proteins with metabolic functions (COG categories C, E, H, I and P), as well as Translation, ribosomal structure and biogenesis (COG category J), are found in the core elements compared to the accessory elements. Again, proteins involved in Defense mechanisms (COG category V) and the Mobilome: phages, transposons and plasmids (COG category X), as well as DNA replication, recombination and repair (COG category L) are more than two-fold over-represented in the proteins unique to a single subspecies or shared by two *E. xiangfangensis* subspecies only. Differences in the functions associated with specific subspecies were also observed with more than two-fold proteins involved in Cell motility (COG category N) and Inorganic ion transport and metabolism (COG category P) specific to *E. xiangfangensis* subsp. *steigerwaltii* (49 and 38 proteins, respectively) compared to subsp. *oharae* (11 and 15 specific proteins, respectively) and subsp. *xiangfangensis* (12 and 14 specific proteins, respectively). By contrast a larger subspecies-specific complement of proteins involved in Lipid transport and metabolism (COG category I) is present in *E. xiangfangensis* subsp. *xiangfangensis* (23 proteins) compared to the subspecies-specific protein complements of subsp. *oharae* (4 proteins) and subsp. *steigerwaltii* (12 proteins).

### Plasmids, phages and transposases are key drivers of diversification

Given the over-representation of proteins in the COG category X in the species- and subspecies-specific pan-genome elements, the genomes were screened for various mobile genomic elements. Plasmid elements were detected using PlasmidFinder v. 2.1 [22] and were found to be prevalent features of the mobilome. On average, 3.44 plasmid replicons are incorporated in the genomes of the 250 *E. hormaechei* complex taxa (Table 2; Supplementary Material 1 Table S6), with no evidence of plasmid sequences in only twenty of the taxa. The largest number of plasmids was observed in *E. hoffmannii* RHBSTW-00316, which incorporates nine complete plasmids. Comparison of the different *E. hormaechei* complex species and subspecies showed that *E. hormaechei* has the highest number of plasmids on average (4.67), while only 2.93 plasmids on average are observed in *E. hoffmannii* strains. Despite this lower number, plasmid DNA contributes the largest proportion of total genomic DNA content and proteins encoded on the genome among the compared taxa, along with *E. xiangfangensis* subsp. *xiangfangensis* (Table 2). On average, 5.65% of the total proteome is plasmid-encoded, with almost one sixth (17.55%) of the proteome of *E. hoffmannii* ECL411 encoded on five distinct plasmids. This provides evidence that plasmids are key drivers of functional diversification in these *Enterobacter* species.

**Table 2:** Mobilome of representative taxa of the *E. hormaechei* complex. The range in number and average number (in brackets) of plasmid, phage, ICE elements and transposable elements are shown. The contribution of plasmid, phage and ICE elements to the total genome size and number of proteins encoded on the genomes are indicated

Species/subspecies	*E. hoffmannii* (50)	*E. hormaechei* (50)	*E. xiangfangensis* (150)	*E. xiangfangensis* subsp. *oharae* (50)	*E. xiangfangensis* subsp. *steigerwaltii* (50)	*E. xiangfangensis* subsp. *xiangfangensis* (50)	*E. quasihormaechei* (1)
**Plasmids (#)**	**0—9 (2.93)**	**0—7 (4.67)**	**0—7 (3.20)**	**0—6 (3.31)**	**0—6 (3.05)**	**0—7 (3.24)**	1*
Plasmid DNA complement (kb)	0—803 (306)	0—592 (237)	0—697 (263)	0—488 (116)	0—650 (328)	0—697 (343)	94
Genomic DNA proportion (%)	0—14.44 (5.96)	0—11.41 (4.60)	0—12.58 (4.57)	0—9.36 (2.14)	0—11.81 (5.56)	0—12.58 (5.99)	2.06
Plasmid protein complement	0—933 (333)	0—653 (256)	0—766 (288)	0—541 (131)	0—690 (355)	0—766 (375)	100
Genomic protein proportion (%)	0—17.55 (6.98)	0—13.31 (5.33)	0—14.49 (5.31)	0—11.05 (2.53)	0—13.29 (6.42)	0—14.49 (6.98)	2.39
**Phage elements (#)**	**0—13 (5.76)**	**1—8 (4.00)**	**0—14 (5.93)**	**1—8 (4.56)**	**0—14 (6.66)**	**2—12 (6.56)**	**4**
Phage DNA complement (kb)	0—519 (232)	22—337 (143)	0—649 (245)	23—322 (175)	0—528 (266)	93—649 (296)	113
Genomic DNA proportion (%)	0—9.33 (4.54)	0.46—6.86 (2.96)	0—12.38 (4.83)	0.47—6.70 (3.63)	0—9.86 (5.07)	1.79—12.38 (5.81)	2.47
Phage protein complement	0—552 (274)	25—426 (167)	0—829 (298)	34—363 (209)	0—666 (322)	115—829 (363)	102
Genomic protein proportion (%)	0—11.08 (5.75)	0.56—9.32 (3.71)	0—19.32 (6.28)	0.78—8.10 (4.67)	0—13.02 (6.54)	2.41—19.32 (7.63)	2.44
**ICE elements (#)**	**0 -2 (0.12)**	**0—1 (0.10)**	**0—2 (0.31)**	**0 -1 (0.06)**	**0—2 (0.62)**	**0—1 (0.26)**	**0**
ICE DNA complement (kb)	0—140 (9)	0—55 (5)	0—272 (27)	0—99 (5)	0—272 (52)	0—137 (24)	0
Genomic DNA proportion (%)	0—2.81 (0.18)	0—1.12 (0.10)	0—4.81 (0.52)	0—2.03 (0.10)	0—4.81 (0.99)	0—2.71 (0.47)	0
ICE protein complement	0—148 (9)	0—46 (4)	0—278 (27)	0—104 (5)	0—278 (54)	0—144 (23)	0
Genomic protein proportion (%)	0—3.15 (0.19)	0—1.04 (0.09)	0—5.18 (0.56)	0—2.40 (0.11)	0—5.18 (1.11)	0—3.11 (0.44)	0
**Transposable elements (#)**	5—136 (51.74)	12—84 (34.88)	1—121 (45.63)	1—112 (39.04)	1—121 (50.48)	5—113 (47.36)	5

PlasmidFinder further classifies detected plasmids on the basis of conserved replicons [22]. This showed that small Col-like plasmids are most prevalent among the compared *E. hormaechei* complex taxa (46.2% of taxa), followed by IncF plasmids (in 42% of taxa), both of which are present in up to three copies per genome (Table 3; Supplementary Material 1 Table S6). The latter plasmids are particularly prevalent in *E. hoffmannii* (58% of taxa) and *E. xiangfangensis* subsp. *xiangfangensis* (70% of taxa) strains. Furthermore, IncH-type plasmids are present in 39.6% of the 250 compared taxa, particularly in strains of *E. hoffmannii* (52% of taxa) and *E. xiangfangensis* subsp. *steigerwaltii* (58% of taxa). Another eleven plasmid types are present in less than 10% of the compared taxa, demonstrating the extensive variability in the plasmid profiles associated with different *E. hormaechei* complex strains.

**Table 3:** Prevalence of different plasmid types in the representative *E. hormaechei* complex species/subspecies. The total number and proportion that each plasmid type contributes to all plasmids in the compared taxa are shown. * indicates those plasmids that are present in more than one copy in a single strain

Plasmid type	*E. hoffmannii*	*E. hormaechei*	*E. xiangfangensis*			Total	Proportion of total (%)
			**subsp.* oharae***	**subsp.* steigerwaltii***	**subsp.* xiangfangensis***		
IncC	1	5	0	1	3	10	4.0
IncF*	29	12	17	12	35	105	42.0
IncH	26	17	3	29	24	99	39.6
IncI	0	0	0	1	0	1	0.4
IncL	2	5	0	2	2	11	4.4
IncM	0	1	0	4	3	8	3.2
IncN	4	3	2	5	2	16	6.4
IncP	0	0	0	1	0	1	0.4
IncR	7	21	19	4	1	52	20.8
IncX	0	8	1	4	12	25	10.0
pKP	2	4	0	6	1	13	5.2
RepA	0	0	0	0	1	1	0.4
RepB	0	2	0	0	1	3	1.2
RepE	1	0	1	0	1	3	1.2
Col*	18	32	22	27	17	116	46.4

PHASTEST [23] predicted a total of 1,377 phage elements on the genomes of the 250 *E. hormaechei* complex strains (on average 5.5% phage elements/genome), with two taxa whose genomes did not integrate any phage elements, while fourteen elements are present in the genome of *E. xiangfangensis* subsp. *steigerwaltii* CPO062 (Table 2; Supplementary Material 1 Table S7). The highest number of phage elements are, on average, integrated in the genomes of *E. xiangfangensis* subsp. *xiangfangensis* (6.6/genome) and *steigerwaltii* (6.7/genome), respectively, while lower numbers are observed in *E. xiangfangensis* subsp. *oharae* (4.6/genome) and *E. hormaechei* (4/genome). Phage-borne proteins contribute 5.7% of the total proteome of the *E. hormaechei* complex, with 16.9% of the *E. xiangfangensis* subsp. *xiangfangensis* OSUCZKPC4-140 proteome derived from phages, indicating that these elements play a key role in genomic diversification. The phage elements showed homology to 99 distinct phages, with 65.7% of these occurring in ten or less of the compared taxa. Two phage elements, Enterobacterial phage mEp390 (NCBI Accession #: NC_019721; 115/250 taxa) and *Erwinia* phage vB_EhrS_59 (NCBI Accession #: NC_048198; 113/250 taxa) were predicted to occur in the genomes of over 100 of the *E. hormaechei* complex taxa.

Integrative and conjugative elements (ICEs) play key roles in the diversification of a broad range of bacterial taxa [24]. Using ICEfinder [25], a total of 58 ICE elements were predicted in the genomes of 56 (22.4%) of the compared *E. hormaechei* complex taxa (Table 2). They are most prevalent in *E. xiangfangensis* subsp. *steigerwaltii* (30/50 strains) and subsp. *xiangfangensis* (13/50 strains), while the genomes of only three *E. xiangfangensis* subsp. *oharae* strains incorporate ICEs. Two strains, *E. hoffmannii* RHBSTW-00040 and *E. xiangfangensis* subsp. *steigerwaltii* Eho-E4 incorporate two ICEs each. While less prolific than plasmid and phage elements (contribute up to only 5.2% of total proteome), ICEfinder [25] nonetheless indicates that they encode several important phenotypes, such as resistance to antibiotics and heavy metals, phage resistance, siderophore biosynthesis and motility (Supplementary Material 1 Table S8), which may contribute to the ecological success of these taxa.

Finally, transposable elements were detected using TnComp_finder [26]. On average, 44.7 transposons were detected on the genomes of the 250 *E. hormaechei* complex (Table 2; Supplementary Material 1 Table S9), with the genomes of three *E. xiangfangensis* strains incorporating only two transposons, while the genomes of *E. hoffmannii* RHBSTW-0040 and ECL411 incorporate 136 transposases. The highest relative abundance of transposase genes occurs in *E. hoffmannii* (51.7/genome), while the lowest occurs in *E. hormaechei* (34.9/genome). Among the *E. xiangfangensis* subspecies, the genomes of the subsp. *steigerwaltii* strains encode on average 11.4 and 3.1 more transposases than subspecies *oharae* and *xiangfangensis*, respectively (Supplementary Material 1 Table S9). Given the extensive variability in transposon profiles among the *E. hormaechei* complex strains and their propensity to contribute to gene disruptions, deletions and horizontal gene acquisition [27], suggests an important role for these elements in both genetic and phenotypic diversification of these taxa.

### Members of the *E. hormaechei* complex have an expansive but relatively conserved set of pathogenicity determinants

The PathogenFinder 1.1 server [28] was used to determine the potential of the 250 *E. hormaechei* complex taxa as human pathogens. This server performs BLASTP searches against a protein family database comprising the proteomes of 372 human pathogens and 513 non-pathogens. The number and type (pathogen specific or non-pathogen specific) of query strain orthologues in the protein family database are used to assign a human pathogen probability score (range 0.0 – 1.0), where a probability score > 0.5 is considered as the threshold for consideration as human pathogen [28]. PathogenFinder predicted that all 250 *E. hormaechei* complex taxa represent potential human pathogens, with an average probability score of 0.768 (range 0.627 – 0.985). Furthermore, the outgroup strain *E. quasihormaechei* WCHEQ12003^T^ was also predicted as human pathogen (probability score: 0.796) and was originally isolated from human sputum in the clinical setting [29]. Among the compared species, the highest average probability score was observed for *E. hoffmannii* (0.785), followed by *E. xiangfangensis* (0.769), while comparison of the *E. xiangfangensis* subspecies showed that subsp. *xiangfangensis* (0.800) displays a substantively higher probability score than the other two subspecies (0.754). PathogenFinder probability scores were slightly higher for clinical isolates (0.769) than for environmental isolates (0.756). However, the highest probability scores were observed for the animal isolates (0.800).

On average, 431 hits for pathogenicity/host interaction determinants in the Pathogen-Host Interaction (PHI) database [30] were predicted on the *E. hormaechei* complex genomes, contributing an average 9.3% of the proteome (Table 4; Supplementary Material 1 Table S10). Higher numbers of hits were observed in the *E. hormaechei* genomes (average: 416.7 hits) compared to *E. hoffmannii* (average 431.9 hits) and *E. xiangfangensis* (average: 435.4 hits). Among the *E. xiangfangensis* subspecies, *E. xiangfangensis* subsp. *steigerwaltii* displayed the highest number of hits (average: 442.9 hits) (Table 4). Clinical isolates displayed slightly more (average: 432 hits) PHI hits than environmental (average: 427.6 hits) and animal isolates (average: 427.6 hits). A total of 478 distinct pathogenicity/host interaction proteins were predicted on the *E. hormaechei* complex genomes, of which 350 (73.2%) were conserved among all 250 compared taxa. Furthermore, 439/478 (91.8%) proteins have representatives in at least one taxon per species, while similarly 430/478 (90.0%) proteins are common to at least one representative of each *E. xiangfangensis* subspecies. This suggests a highly conserved proteome involved in pathogenicity and host interaction. However, some PHI aspects are specific to the different taxonomic groups. All *E. hoffmannii* and *E. xiangfangensis* strains incorporate a locus (*srlAEBDMR*) involved in sorbitol transport and metabolism, which is absent from *E. hormaechei* strains. This locus plays a role in lettuce leaf colonisation in *Salmonella enterica* serovar Typhimurium 14028s [31]. The salmochelin siderophore biosynthetic genes *iroBCDEN* contribute to virulence in extra-intestinal pathogenic *Escherichia coli* and *S. enterica* serovar Typhi in human and animal hosts [32, 33]. The latter genes are unique among the *E. hormaechei* complex in all 50 compared *E. xiangfangensis* subsp. *steigerwaltii* strains.

**Table 4:** Pathogenicity profile of the representative *E. hormaechei* complex species/subspecies. The range and average probability values of each strain being a pathogen as calculated using PathogenFinder [28] are shown. The range and average number of hits against the Pathogen-Host Interaction (PHI) database [30] and secretion systems as predicted using MacSyFinder v. 2 [34] with the TXSScan model [35] are indicated

Species/subspecies	Predicted Human Pathogen	Pathogen Probability	Input Proteome Coverage	PHI-Base Hits	Secretion systems
*E. hoffmannii* (50)	50	0.689—0.985 (0.785)	0.67—1.86 (1.00)	421—440 (431.9)	12—16 (13.96)
*E. hormaechei* (50)	50	0.627—0 .876 (0.749)	0.75—1.77 (0.96)	402—426 (416.7)	8—14 (11.38)
*E. xiangfangensis* (150)	150	0.692—0.901 (0.769)	0.61—1.73 (0.92)	413—460 (435.4)	9—17 (12.57)
subsp. *oharae* (50)	50	0.714—0.901 (0.754)	0.65—1.72 (0.82)	413—435 (429.0)	10—14 (11.5)
subsp. *steigerwaltii* (50)	50	0.692—0.796 (0.753)	0.66—1.23 (0.83)	426—460 (442.9)	9 -16 (12.94)
subsp. *xiangfangensis* (50)	50	0.701—0.898 (0.800)	0.61 -1.73 (1.11)	422—452 (434.3)	11—17 (13.28)
*E. quasihormaechei* (1)	1	0.796	0.77	414	12

Key to microbial pathogen-host interactions are secretion systems, which allow the bacterial pathogen to secrete a range of effectors that can target host cells, causing disease or subverting host cell responses [36]. Given their key roles in pathogen-host interaction, pathogenesis and virulence the secretion systems in the *E. hormaechei* complex taxa were profiled using MacSyFinder v. 2 [34]. The genomes of these taxa encode Type I, II, IV, V and VI secretion systems, along with flagellar systems, while Type III, VII, VIII, IX secretion systems were not evident (Table 4; Supplementary Material 1 Table S10). Type I secretion systems (T1SSs) comprise three proteins, including an inner membrane ABC transporter, a periplasmic adaptor and an outer membrane factor, which secrete a range of proteins such repeat-in-toxins (RTX) exoproteins, Ca^2+^-binding proteins, class II microcins and lipoproteins [37]. On average, three T1SSs are encoded on the genomes of the *E. hormaechei* complex comparators, although the genome of one strain, *E. hormaechei* ECL-14–60 does not contain any T1SS loci, and the genomes of 39.2% of the compared strains incorporate four such loci. The multimeric Type II secretion system (T2SS) is involved in secretion of a range of different substrates including lipases, proteases and digestive enzymes which are key to virulence in many Gram-negative bacterial pathogens [38]. T2SSs were identified in the genome of 240 *E. hormaechei* complex, being absent in four and five *E. xiangfangensis* subsp. *steigerwaltii* and subsp. *xiangfangensis* strains, respectively (Supplementary Material 1 Table S10). Functions of the Type IV secretion system (T4SS) include effector protein secretion into eukaryotic target cells, conjugation and release or uptake of DNA from/to the environment [39]. MacSyFinder screened for protein secretion T4SSs (pT4SS) [35] and, while the majority of strains lacked pT4SSs (179/250; 71.6%), a single pT4SS was identified in 51 strains, two pT4SSs in 16 strains, and three pT4SSs in four *E. hormaechei* strains. Type V secretion systems (T5SSs), or autotransporters, have been subdivided into simple autotransporters (T5aSS), two-partner systems (T5bSS) and trimeric systems (T5cSS). They secrete extracellular passenger domains with virulence (e.g. proteases, lipases, haemolysins), adhesion, intracellular motility and immune evasion functions [40]. Between two and five T5aSSs are encoded on the genomes of the 250 compared *E. hormaechei* complex strains, while a single T5bSS is encoded on the genomes of 49/50 *E. hormaechei* and 25/50 *E. xiangfangensis* subsp. *xiangfangensis* strains (Supplementary Material 1 Table S10). By contrast the T5cSS is more conserved, being present in 231/250 compared strains, with three copies in *E. hormaechei* 98Q0113. Type VI secretion systems (T6SSs) are primarily known for their role in killing competitor bacteria, but also secrete effector proteins involved in virulence against fungal, animal and human hosts [41]. No evidence of T6SSs was found in the genomes of only two *E. hormaechei* strains, namely BSI106 and UCI-CRE132. By contrast 152 strains (60.8%) encode two T6SS on their genomes, while fourteen strains, including five strains of *E. hoffmannii* and nine strains of *E. xiangfangensis* (all three subspecies) encode three T6SS (Supplementary Material 1 Table S10).

Aside from the primary peritrichous flagellar system (*flag-1* locus) present in all comparator *E. hormaechei* complex taxa, an additional flagellar biosynthetic locus was observed in 29 *E. xiangfangensis* subsp. *steigerwaltii* and *E. quasihormaechei* WCHEQ12003^T^ (Supplementary Material 1 Table S10). This locus shows synteny with the *flag-3a* locus, predicted to encode a peritrichous flagellum [42]. While the function of this flagellar system remains unknown, the dual presence of peritrichous flagellar systems has been postulated to serve a role in alternative modes of transport (e.g. swimming versus swarming), alternate expression to avoid recognition and response in both plant and animal hosts towards the highly immunogenic flagellin proteins, or potential roles other than motility, such as secretion of virulence factors [42]. Type IV pili, aside from a role in motility across surfaces, also contribute towards surface sensing, biofilm formation, protein secretion and virulence [43]. MacSyFinder [34] predicted at least one Type IV pilus locus per genome, while two strains *E. hormaechei* strain 205,871 and *E. xiangfangensis* subsp. *steigerwaltii* NCTC 11593 encode three such Type IV pili (Supplementary Material 1 Table S10).

### The *E. hormaechei* complex displays a broad and variable resistome driving multi-drug resistance

The emergence of antibiotic resistance in *E. hormaechei* strains is of mounting concern, given the increasing recognition of these microorganisms as important nosocomial and opportunistic pathogens [2, 3]. The propensity of taxa in this complex to acquire various mobile genetic elements as highlighted above have led to their adaptation to the hospital environment and the evolution of multidrug resistance (MDR) phenotypes [2, 3]. The putative antibiotic resistance phenotypes for the 250 comparator *E. hormaechei* complex taxa were predicted using ResFinder v. 4.6 [44]. This server predicts resistance phenotypes on the basis of the identification of acquired genes and chromosomal mutations in the genome [44]. On average the 250 strains were predicted to display resistance to 23.4 (range: 11 – 45) of the 92 (25.4%) evaluated antibiotics (Table 5; Supplementary Material 1 Table S11). By contrast, *E. quasihormaechei* WCHEQ12003^T^ is predicted to be resistant to only 11/92 antibiotics. Comparison of resistance at the species level showed relatively similar average numbers of antibiotics to which each species was resistant (Table 5). More defined differences were observed among the *E. xiangfangensis* subspecies where, while on average subsp. *xiangfangensis* and *steigerwaltii* strains were predicted to display resistance towards 26.9 antibiotics, subspecies *oharae* strains are putatively resistant to only 15.8 antibiotics on average (Table 5). This could correlate to the smaller genome sizes and lower number of mobile genetic elements such as phages, ICE elements and transposases, in the latter subspecies. Comparison of the observed resistance profiles on the basis of source of isolation (Supplementary Material 1 Table S2) showed some marked differences. Environmental isolates are predicted to be resistant to an average 16.1 of the evaluated antibiotics, while clinical isolates are predicted to be resistant to 24.6 of the 92 antibiotics. By contrast, animal isolates showed simulated resistance phenotypes to 29.5 antibiotics. It should be noted that ResFinder predicted antibiotic resistance phenotypes on the basis of the genotype only. The presence of specific antibiotic resistance genes and chromosomal mutations provide only an in silico prediction of resistance, and phenotypic assays should be performed to confirm the true antibiotic resistance phenotype of the studied *E. hormaechei* complex taxa.

**Table 5:** Resistome profile of the representative *E. hormaechei* complex species/subspecies. ResFinder v. 4.6 [44] was used to predict resistance phenotypes and the average and range of antibiotics, as well as antibiotic resistance classes, each species/subspecies is resistant to was recorded. The proportion of strains per species/subspecies resistant to antibiotics in three or more classes, and hence defined as multidrug resistant [45] is reported. Antibacterial biocide and heavy metal resistance proteins were profiled using the BacMet database [46] and the average number and range of orthologue hits are indicated

Species/subspecies	Antibiotics resistant to	Antibiotic classes resistant to	Multidrug resistant	BacMet Hits
*E. hoffmannii* (50)	22.72 (11 -45)	5.26 (1—9)	39 (78%)	108—169 (146.58)
*E. hormaechei* (50)	24.68 (11—44)	4.86 (1—10)	34 (68%)	108—156 (136.08)
*E. xiangfangensis* (150)	23.16 (11—42)	5.01 (1—10)	106 (71%)	104—169 (139.20)
subsp. *oharae* (50)	15.76 (11—41)	2.56 (1—10)	20 (40%)	108—169 (132.94)
subsp. *steigerwaltii* (50)	26.4 (12—42)	6.16 (2—10)	41 (82%)	104—168 (145.92)
subsp. *xiangfangensis* (50)	27.32 (12—40)	6.32 (2—10)	45 (90%)	108—168 (138.74)
*E. quasihormaechei* (1)	11	1	0 (0%)	107

Antibiotic resistance genes (ARGs) were derived from the ResFinder v. 4.6 [44] output. A total of 2,335 were predicted across the 250 *E. hormaechei* complex taxa, showing homology to 104 distinct ARGs (Table 6; Supplementary Material 1 Table S12). Only a restricted proportion (500 ARGs; 21.4% of total) are localised on the chromosome, 62 ARGs were found on unplaced contigs, while 1,773 ARGs (75.9% of total) are predicted to reside on plasmids. This highlights the importance of these mobile genetic elements in the dissemination of antibiotic resistance. ARGs are particularly prevalent on IncH-type plasmids, with 50.6% of plasmid-encoded on these elements, while 277 ARGs are harboured on IncF-type plasmids (Supplementary Material 1 Table S12). However, prediction of the specific plasmid element harbouring specific ARGs is complex as some ARGs are present on diverse plasmid replicons. Only two ARGs are exclusively found on the chromosome, namely *blaACT* and *fosA*, which code for a broad-spectrum class C beta-lactamase and fosfomycin resistance protein, respectively. These are the most prevalent ARGs, with *blaACT* present in all compared *E. hormaechei* complex taxa and *fosA* present in 143/250 taxa. The latter gene has been lost from the genomes of all compared *E. hormaechei* and *E. xiangfangensis* subsp. *oharae* strains. Other ARGs, which are plasmid-borne, are more restricted in distribution, with 18 ARGs restricted to single strains (Table 6; Supplementary Material 1 Table S12). This includes three genes (*tmexC2, tmexD2, tOprJ2*) comprising a tetracycline multidrug resistance system which is restricted to the IncF-type plasmid of *E. hoffmannii* JH25 and the extended-spectrum beta-lactamase gene *blaVEB-3* and carbapenamase gene *blaCARB-2* in *E. xiangfangensis* subsp. *steigerwaltii* C45 (IncF-type plasmid) and *E. hormaechei* 35666 (Unclassified plasmid), respectively. The highest number of ARGs were predicted in E. *xiangfangensis* subsp. *steigerwaltii* strains CPO51 and va18651, with 32 distinct ARGs/genome. It should be noted that in both cases, several ARGs are present in multiple copy number on the plasmids, with 24 distinct ARGs encoded on the genomes. However, the role of these ARGs will need to be confirmed using phenotypic assays. By contrast, a single chromosomal ARG (*blaACT*) is found in 37/250 strains, while 29/250 strains only harbour *blaACT* and *fosA* on their chromosomes, again highlighting the role of plasmids in the antibiotic resistance repertoires of *E. hormaechei* complex strains.

**Table 6:** Prediction of ARGs on the genomes of the *E. hormaechei* complex species/subspecies. ARGs were predicted using ResFinder v. 4.6 [44] and the presence/absence of each distinct ARG was collated

Gene	Class	Antibiotic phenotype resistance	NCBI Accession	*E. hoffmannii*	*E. hormaechei*	*E. xiangfangensis*			Total
			**best hit**			**subsp. *oharae***	**subsp. *steigerwaltii***	**subsp. *xiangfangensis***	
*aac(3)-I*	Aminoglycoside	gentamicin, astromicin, fortimicin	AJ877225	0	0	0	1	0	1
*aac(3)-Ib*	Aminoglycoside	gentamicin, astromicin, fortimicin	L06157	1	0	1	0	1	3
*aac(3)-IIa*	Aminoglycoside	gentamicin, tobramycin	CP023555	3	2	11	2	7	25
*aac(3)-IId*	Aminoglycoside	gentamicin, tobramycin, dibekacin, netilmicin, apramycin, sisomicin	EU022314	3	3	1	9	6	22
*aac(6')-Ib*	Aminoglycoside	tobramycin, amikacin	M21682	0	3	0	1	8	12
*aac(6')-lb-cr*	Aminoglycoside	tobramycin, amikacin, dibekacin, netilmicin, sisomicin, fluoroquinolone, ciprofloxacin	DQ303918	5	16	3	11	9	44
*aac(6')-lb-Hangzhou*	Aminoglycoside	tobramycin, amikacin	FJ503047	0	0	0	2	2	4
*aac(6')-lb3*	Aminoglycoside	tobramycin, amikacin	X60321	7	8	0	16	7	38
*aac(6')-lI*	Aminoglycoside	tobramycin, amikacin	U13880	1	2	1	1	1	6
*aac(6')-IIc*	Aminoglycoside	gentamicin, tobramycin	NC_012555	19	1	1	19	4	44
*aadA1*	Aminoglycoside	streptomycin, spectinomycin	JX185132	7	15	13	10	20	65
*aadA2*	Aminoglycoside	streptomycin, spectinomycin	JQ364967	5	6	3	8	13	35
*aadA5*	Aminoglycoside	streptomycin, spectinomycin	AF137361	0	0	0	0	2	2
*aadA16*	Aminoglycoside	streptomycin, spectinomycin	EU675686	1	8	1	1	1	12
*aadA22*	Aminoglycoside	streptomycin, spectinomycin	AJ809407	1	1	1	1	2	6
*aadB*	Aminoglycoside	gentamicin, tobramycin	X04555	4	6	0	4	5	19
*aph(3')-Ia*	Aminoglycoside	kanamycin, neomycin, lividomycin, paromomycin, ribostamycin	V00359	2	5	1	4	11	23
*aph(3')-Ib*	Aminoglycoside	kanamycin	AJ744860	0	0	0	1	0	1
*aph(3'')-Ib*	Aminoglycoside	streptomycin	AF024602	5	11	3	22	29	70
*aph(3')-IIa*	Aminoglycoside	kanamycin, neomycin	V00618	0	0	0	1	0	1
*aph(3)-VI*	Aminoglycoside	amikacin	KC170992	0	0	0	0	2	2
*aph(3)-VIb*	Aminoglycoside	gentamicin, amikacin, kanamycin, neomycin, paromomycin, ribostamycin, butirosin	AJ627643	1	0	0	0	1	2
*aph(6)-Ic*	Aminoglycoside	streptomycin	X01702	0	0	0	1	0	1
*aph(6)-Id*	Aminoglycoside	streptomycin	M28829	5	11	14	22	29	81
*armA*	Aminoglycoside	gentamicin, tobramycin, amikacin, isepamicin, netilmicin	AY220558	0	2	2	2	2	8
*rmtB*	Aminoglycoside	gentamicin, tobramycin, amikacin, isepamicin, kanamycin, sisomicin, arbekacin	AB103506	4	11	3	8	4	30
*rmtC*	Aminoglycoside	gentamicin, tobramycin, amikacin, isepamicin, kanamycin, sisomicin, arbekacin	AB194779	3	0	0	1	0	4
*catA1*	Amphenicol	chloramphenicol	V00622	50	50	50	50	50	250
*catA2*	Amphenicol	chloramphenicol	X53796	0	1	0	0	0	1
*catB2*	Amphenicol	chloramphenicol	AF047479	1	0	0	3	4	8
*catB3*	Amphenicol	chloramphenicol	AJ009818	5	13	3	3	10	34
*cmlA1*	Amphenicol	chloramphenicol	M64556	2	2	0	2	2	8
*floR*	Amphenicol	chloramphenicol, florfenicol	AF118107	0	1	0	1	0	2
*blaVEB-3*	B-lactam	unknown beta-lactam	AY536519	16	0	0	13	2	31
*blaCARB-2*	B-lactam (Class A)	amoxicillin, ampicillin, piperacillin	M69058	1	2	0	9	3	15
*blaCTX-M-9*	B-lactam (Class A)	amoxicillin, ampicillin, cefepime, cefotaxime, ceftazidime, piperacillin, aztreonam, ticarcillin, ceftriaxone	AF174129	0	0	0	1	1	2
*blaCTX-M-15*	B-lactam (Class A)	amoxicillin, ampicillin, cefepime, cefotaxime, ceftazidime, piperacillin, aztreonam, ticarcillin, ceftriaxone	Y044436	4	5	0	4	12	25
*blaLAP-2*	B-lactam (Class A)	amoxicillin, ampicillin, piperacillin, ticarcillin, cephalotin	EU159120	0	8	1	2	2	13
*blaSFO-1*	B-lactam (Class A)	amoxicillin, ampicillin, cefotaxime, cefoxitin, ceftazidime, piperacillin, ticarcillin	AB003148	4	11	2	3	14	34
*blaSHV-12*	B-lactam (Class A)	amoxicillin, ampicillin, cefepime, cefotaxime, ceftazidime, piperacillin, aztreonam, ticarcillin, ceftriaxone	KF976405	4	9	2	10	9	34
*blaSHV-30*	B-lactam (Class A)	amoxicillin, ampicillin, cefepime, cefotaxime, ceftazidime, piperacillin, aztreonam, ticarcillin, ceftriaxone	AY661885	0	3	0	1	8	12
*blaTEM-1A/B*	B-lactam (Class A)	amoxicillin, ampicillin, piperacillin, ticarcillin, cephalothin	HM749966	2	3	2	2	0	9
*blaTEM-2*	B-lactam (Class A)	amoxicillin, ampicillin, piperacillin, ticarcillin, cephalothin	X54606	1	5	0	2	2	10
*blaKPC-2/3/4/6*	B-lactam (Class A; Group 2f)	amoxicillin, amoxicillin + clavulanic acid, ampicillin, ampicillin + clavulanic acid, cefepime, cefotaxime, cefoxitin, ceftazidime, ertapenem, imipenem, meropenem, piperacillin, piperacillin + tazobactam, aztreonam, ticarcillin, ticarcillin + clavulanic acid	AY034847	0	0	0	0	3	3
*blaACC-1*	B-lactam (Class C)	amoxicillin, amoxicillin + clavulanic acid, ampicillin, ampicillin + clavulanic acid, cefotaxime, cefoxitin, ceftazidime, piperacillin, piperacillin + tazobactam, ticarcillin, ticarcillin + clavulanic acid	HG530658	1	0	0	1	2	4
*blaACT-5/7/14/15/16*	B-lactam (Class C)	amoxicillin, amoxicillin + clavulanic acid, ampicillin, ampicillin + clavulanic acid, cefotaxime, cefoxitin, ceftazidime, piperacillin, piperacillin + tazobactam, ticarcillin, ticarcillin + clavulanic acid	FJ237369	3	3	1	12	15	34
*blaDHA-1*	B-lactam (Class C)	amoxicillin, amoxicillin + clavulanic acid, ampicillin, ampicillin + clavulanic acid, cefotaxime, cefoxitin, ceftazidime, piperacillin, piperacillin + tazobactam, ticarcillin, ticarcillin + clavulanic acid	Y16410	0	2	0	0	2	4
*blaFOX-5*	B-lactam (Class C)	amoxicillin, amoxicillin + clavulanic acid, ampicillin, ampicillin + clavulanic acid, cefotaxime, cefoxitin, ceftazidime, piperacillin, piperacillin + tazobactam, ticarcillin, ticarcillin + clavulanic acid	AY007369	9	19	3	24	30	85
*blaOXA-1*	B-lactam (Class D)	amoxicillin, amoxicillin + clavulanic acid, ampicillin, ampicillin + clavulanic acid, cefepime, piperacillin, piperacillin + tazobactam	HQ170510	0	0	10	1	0	11
*blaOXA-9*	B-lactam (Class D)	amoxicillin, ampicillin	KQ089875	0	0	0	1	0	1
*blaOXA-10*	B-lactam (Class D)	amoxicillin, ampicillin, piperacillin, piperacillin + tazobactam, aztreonam	J03427	5	8	1	5	2	21
*blaOXA-48*	B-lactam (Class D)	amoxicillin, amoxicillin + clavulanic acid, ampicillin, ampicillin + clavulanic acid, imipenem, meropenem, piperacillin, piperacillin + tazobactam	AY236073	0	1	0	0	0	1
*blaOXA-129*	B-lactam (Class D)	unknown beta-lactam	FJWZ01000025	2	6	0	5	10	23
*blaIMP-1*	B-lactam (Subclass B1)	amoxicillin, amoxicillin + clavulanic acid, ampicillin, ampicillin + clavulanic acid, cefepime, cefixime, cefotaxime, cefoxitin, ceftazidime, ertapenem, imipenem, meropenem, piperacillin, piperacillin + tazobactam	EF027105	2	1	1	13	8	25
*blaIMP-4*	B-lactam (Subclass B1)	amoxicillin, amoxicillin + clavulanic acid, ampicillin, ampicillin + clavulanic acid, cefepime, cefixime, cefotaxime, cefoxitin, ceftazidime, ertapenem, imipenem, meropenem, piperacillin, piperacillin + tazobactam	AF244145	1	0	0	0	0	1
*blaIMP-8*	B-lactam (Subclass B1)	amoxicillin, amoxicillin + clavulanic acid, ampicillin, ampicillin + clavulanic acid, cefepime, cefixime, cefotaxime, cefoxitin, ceftazidime, ertapenem, imipenem, meropenem, piperacillin, piperacillin + tazobactam	DQ845788	4	10	2	14	10	40
*blaNDM-1/5/7*	B-lactam (Subclass B1)	amoxicillin, amoxicillin + clavulanic acid, ampicillin, ampicillin + clavulanic acid, cefepime, cefixime, cefotaxime, cefoxitin, ceftazidime, ertapenem, imipenem, meropenem, piperacillin, piperacillin + tazobactam, temocillin, ceftazidime + avibactam	FN396876	0	1	0	1	0	2
*blaVIM-1/4*	B-lactam (Subclass B1)	amoxicillin, amoxicillin + clavulanic acid, ampicillin, ampicillin + clavulanic acid, cefepime, cefixime, cefotaxime, cefoxitin, ceftazidime, ertapenem, imipenem, meropenem, piperacillin, piperacillin + tazobactam	Y18050	1	2	13	1	4	21
*dfrA1*	Folate pathway antagonist	trimethoprim	AF203818	0	0	0	0	1	1
*dfrA8*	Folate pathway antagonist	trimethoprim	U10186	1	3	2	1	5	12
*dfrA12*	Folate pathway antagonist	trimethoprim	AM040708	6	11	3	6	21	47
*dfrA14*	Folate pathway antagonist	trimethoprim	KF921535	7	0	0	0	0	7
*dfrA15*	Folate pathway antagonist	trimethoprim	AF221900	0	0	0	2	3	5
*dfrA16*	Folate pathway antagonist	trimethoprim	AF174129	0	0	0	0	2	2
*dfrA17*	Folate pathway antagonist	trimethoprim	FJ460238	3	2	1	12	7	25
*dfrA19*	Folate pathway antagonist	trimethoprim	EU855687	0	0	0	0	3	3
*drfA21*	Folate pathway antagonist	trimethoprim	AY552589	0	1	0	0	0	1
*dfrA25*	Folate pathway antagonist	trimethoprim	DQ267940	2	8	1	1	1	13
*dfrA27*	Folate pathway antagonist	trimethoprim	FJ459817	1	0	0	0	0	1
*dfrB1*	Folate pathway antagonist	trimethoprim	U36276	0	2	0	0	0	2
*dfrB3*	Folate pathway antagonist	trimethoprim	X72585	3	1	1	6	4	15
*sul1*	Folate pathway antagonist	sulfamethoxazole	U12338	1	5	1	1	2	10
*sul2*	Folate pathway antagonist	sulfamethoxazole	AY034138	49	0	0	45	49	143
*sul3*	Folate pathway antagonist	sulfamethoxazole	AJ459418	0	0	0	0	2	2
*fosA*	Fosfomycin	fosfomycin	M85195	1	1	0	0	0	2
*fosA3*	Fosfomycin	fosfomycin	AB522970	0	1	1	0	0	2
*fosA5*	Fosfomycin	fosfomycin	EU195449	0	0	0	0	1	1
*bleO*	Glycopeptide	bleomycin	AF051917	0	0	0	0	3	3
*lnu(F)*	Lincosamide	lincomycin	EU118119	5	13	1	11	8	38
*lnu(G)*	Lincosamide	lincomycin	KX470419	4	4	2	3	0	13
*ere(A)*	Macrolide	erythromycin	AF099140	4	4	2	3	0	13
*mph(A)*	Macrolide	erythromycin, azithromycin, spiramycin, telithromycin	D16251	2	7	2	6	7	24
*mph(E)*	Macrolide	erythromycin	DQ839391	0	1	0	0	0	1
*msr(E)*	Macrolide/Streptagramin a	erythromycin, azithromycin, quinupristin, pristinamycin ia, virginiamycin s	FR751518	3	10	2	2	9	26
*mcr10*	Polymyxin	colistin	MN179494	1	3	0	9	2	15
*qnrA1*	Quinolone	ciprofloxacin	AY070235	2	1	0	2	2	7
*qnrA7*	Quinolone	ciprofloxacin	GQ463707	16	4	1	12	1	34
*qnrB1*	Quinolone	ciprofloxacin	DQ351241	0	2	0	0	0	2
*qnrB2*	Quinolone	ciprofloxacin	DQ351242	1	1	0	0	0	2
*qnrB4*	Quinolone	ciprofloxacin	DQ303921	2	13	2	6	8	31
*qnrB6*	Quinolone	ciprofloxacin	EF523819	0	0	0	1	1	2
*qnrB7*	Quinolone	ciprofloxacin	EU043311	2	0	0	0	0	2
*qnrB19*	Quinolone	ciprofloxacin	EU432277	34	29	15	38	27	143
*qnrS1*	Quinolone	ciprofloxacin	AB187515	2	9	2	9	20	42
*ARR-3*	Rifamycin	rifampicin	JF806499	0	1	1	0	0	2
*tet(A)*	Tetracycline	tetracycline, doxycycline	AJ517790	5	17	1	7	13	43
*tet(B)*	Tetracycline	tetracycline, doxycycline, minocycline	AP000342	16	2	2	13	2	35
*tet(C)*	Tetracycline	tetracycline, doxycycline	AF055345	0	0	1	0	0	1
*tet(D)*	Tetracycline	tetracycline, doxycycline	AF467077	2	1	2	11	13	29
*tet(G)*	Tetracycline	tetracycline, doxycycline	AF133140	0	0	0	3	2	5
*tet(X4)*	Tetracycline	tetracycline, doxycycline, minocycline, tigecycline	MK134376	0	0	0	0	1	1
*tmexC2*	Tetracycline	tetracycline, doxycycline, minocycline, tigecycline	MN175502	1	0	0	0	0	1
*tmexD2*	Tetracycline	tetracycline, doxycycline, minocycline, tigecycline	MN175502	1	0	0	0	0	1
*tOprJ2*	Tetracycline	tetracycline, doxycycline, minocycline, tigecycline	MN175502	1	0	0	0	0	1

Multidrug resistant bacteria are defined as those bacteria that are resistant to antibiotics in three or more antimicrobial classes [45]. By this definition, 179/250 (71.6%) of the *E. hormaechei* complex taxa are predicted to be multidrug resistant on the basis of in silico data (Table 5; Supplementary Material 1 Table S11). *E. hoffmannii* was predicted to display the broadest antibiotic class resistance, putatively resistant to antibiotics belonging to an average of 5.3 of the twenty-one classes evaluated by ResFinder v. 4.6 [44]. However, the lower value for *E. xiangfangensis* is again skewed by *E. xiangfangensis* subsp. *oharae*, where strains are resistant to antibiotics in an average 2.6 classes, while subspecies *steigerwaltii* and *xiangfangensis* are predicted to be resistant to a combined average of 6.2 antibiotic classes (Table 5). Further, while 40% of *E. xiangfangensis* subsp. *oharae* are predicted to be multidrug resistant,, 82% and 90% of *E. xiangfangensis* subsp. *steigerwaltii* and *xiangfangensis*, respectively are putatively multidrug resistant. Resistance to only a single antibiotic class, the beta-lactams, is predicted for all 250 comparator taxa, while resistance to aminoglycosides and folate pathway resistance antibiotics was predicted in 69.6% and 68% of the strains, respectively. Conversely, resistance to tigecycline (tetracycline class) and colistin (polymyxin class) were predicted to occur in only 2/250 (*E. hoffmannii* JH25 and *E. xiangfangensis* subsp. *xiangfangensis* GX4-8L) and 3/250 strains (three strains of *E. xiangfangensis* subsp. *xiangfangensis*), respectively. Despite their low prevalence, the predicted resistance to these antibiotics is of concern, as these are last-line antibiotics, used when all other treatment options have failed [2].

*E. hoffmannii* Eh1 (45/92 antibiotics; 8 classes) and *E. hormaechei* CF4 (44/92 antibiotics; 9 classes) were predicted to be resistant to the greatest number of distinct antibiotics. These strains were isolated from a clinical specimen and from a goose, respectively (Supplementary Material 1 Tables S2 and S11). Again, the true resistance phenotypes for these taxa needs to be validated beyond this initial in silico prediction. Mapping of potential antibiotic resistance against the sources of isolation of the strains showed limited correlation, with 97/114 (85.1%) of strains with putative resistance to ≥ 25 of the evaluated antibiotics being of clinical origin, 10/114 (8.8%) of animal origin and 7/114 (6.1%) of environmental origin. This likely rather reflects the predominance of clinical isolates (78.8%) in the subset of 250 strains. ResFinder [44] also screened the strains for potential resistance against common disinfectants. Resistance to quaternary ammonium compounds, frequently used as antiseptics and disinfectants in both the clinical and domestic setting [47], is widespread among the *E. hormaechei* complex strains, occurring in 55.6% of the studied taxa and represented across all three species and *E. xiangfangensis* subspecies (Supplementary Material 1 Table S11). Although more sparse, 15/250 strains are also predicted to be resistant to formaldehyde. This suggests adaptive evolution of *E. hormaechei* complex strains towards a broad range of environmental stressors.

Given the frequent isolation of *E. hormaechei* complex strains from the natural environment, their ability to deal with various environmental stressors was further elucidated by comparing the proteomes against the BacMet database v. 2.0, a resource for the identification of antibacterial biocide- and metal-resistance genes [46]. Orthologues of a total 169 distinct resistance genes were identified on the genomes of the 250 *E. hormaechei* complex strains (Table 5; Supplementary Material 1 Table S13). Of these, 55.6% are core to all 250 taxa, highlighting the broader biocide and metal resistance of these species. This includes genes conferring resistance to environmental contaminants such as acriflavine, phenol, cyclohexane, sodium dodecyl sulphate, hydrogen peroxide and hydrochloric acid as well as metals, such as arsenic, tungsten and copper. A further 33.7% of the resistance genes are represented at least once among the 50 taxa of each species and subspecies (Supplementary Material 1 Table S13). These more restricted genes encode phenotypes such as resistance to iron, nickel, silver and mercury.

## Discussion

The *Enterobacter hormaechei* complex comprises pathogens of increasing concern, being linked to mounting cases of hospital-acquired infections and outbreaks [3]. This is exacerbated by their environmental persistence and their ability to acquire antibiotic resistance genes, leading to the development of multidrug resistance phenotypes [2, 3]. The comparative genomic analysis in this study highlights the extensive genetic diversity among members of this complex, evidenced by their open pan-genomes, as well as the phylogenomic analysis, which supports the consideration of the *E. hormaechei* complex as comprising of three species, *E. hormaechei*, *E. hoffmannii* and *E. xiangfangensis*, with the latter incorporating three distinct subspecies, *oharae, steigerwaltii* and *xiangfangensis*, respectively [1, 7]. Given the emerging role of the *E. hormaechei* complex taxa as antibiotic resistant human pathogens, the accurate identification at the species and subspecies level is pertinent, to elucidate differences in their clinical manifestations and for targeted therapeutic strategies. As such, future work should elucidate the phenotypic characteristics to support some of the identified genomic differences for effective species and subspecies description.

Notable in this study was that, while there were a large number of proteins specific to each species/subspecies, few were conserved in all taxa of these species/subspecies, highlighting the variability even within species/subspecies. This can be attributed to the broad range of mobile genetic elements, including various distinct plasmids, phages and ICE elements. These were not exclusive to a single species/subspecies, but rather occurred patchily in single or several strains of each species and/or subspecies, potentially being shared during colocalization events in the clinical setting or natural environment. These elements may further contribute to the ability of *E. hormaechei* complex taxa to survive and persist in various environments [2, 3]. This is further evidenced by the wide-spread presence of genes conferring resistance to environmental pollutants such as heavy metals and quaternary ammonium compounds. More extensive research on specific mobile elements, such as plasmids and ICEs, is required to shed light on their roles in the environmental persistence, disease development and manifestation and as potential therapeutic targets against *E. hormaechei* complex taxa. A recent study identified an IncHI2 “superplasmid” in *E. hormaechei* C210017 (identified as *E. xiangfangensis* subsp. *steigerwaltii* here), which harboured colistin and carbapenem resistance (*mcr-9* and *bla*_KPC-2_), which was further shown to be present in 131/178 of the *E. hormaechei* strains in the study [48].

While adapted for environmental persistence, PathogenFinder [28] predicted that all 250 *E. hormaechei* complex taxa, regardless of species/subspecies delineation, are human pathogens. This was supported by the expansive pathogenome, which was relatively conserved among the compared strains. However, differences were noted in the secretion systems encoded on the *E. hormaechei* complex genomes. Type III secretion systems were globally absent from the compared taxa, and analysis of the related pathogen *E. cloacae* showed it to be a rare trait, present in only 27% of strains of the latter species [49]. However, T1SS, T2SS, T4SS, T5SS and T6SS were present among the compared taxa, albeit in variable numbers and different prevalences among the *E. hormaechei* complex species and subspecies. There is a dearth of knowledge on the specific functions of these pathogenicity factors in the *E. hormaechei* persistence and future research should focus on elucidating their roles in disease.

Of grave concern was the broad scale multidrug resistance predicted among the *E. hormaechei* complex, with almost three quarters of the compared strains predicted to be resistant to antibiotics in three or more classes, highlighting the urgent need for responsible antibiotic use for these important pathogens. Prior research has demonstrated resistance to a broad range of antibiotics including ß-lactams, cephalosporins (first, second and third generation), aminoglycosides, sulfonamides, carbapenems and combination antibiotics (e.g. piperacillin and tazobactam) in *E, hormaechei* strains isolated from clinical and environmental settings, worldwide [2, 50–56]. These studies have primarily focused on individual or small sets of strains. The comparative genomic analysis presented here underscores the broad scale antibiotic resistance predicted for the *E. hormaechei* complex species and subspecies. However, the genomes only allowed in silico predictions of antibiotic resistance genes and further phenotypic validation of the true resistance phenotypes is required.

Disconcertingly, more than half of the compared strains are predicted to be resistant to last-line carbapenems such as meropenem and imipenem, while resistance to colistin (3/250) and tigecycline (2/250) appears to be emerging, aligning with recent findings on members of the *E. hormaechei* complex [2, 50, 51, 53]. Antibiotic resistance could be linked to 104 distinct ARGs, which are primarily harboured on several different plasmids, highlighting the role of these mobile genetic elements in resistance dissemination [48]. As with the genetic diversity at the whole genome level, ARG prevalence and resistance to specific antibiotics transcends the species and subspecies boundaries, which adds to the complexity of selecting suitable antimicrobial treatments for specific pathogenic strains. This highlights the need for robust genomic surveillance mechanisms to develop effective control measures for infections caused by members of the *E. hormaechei* complex.

## Conclusions

Comparative genomic and pan-genome analysis of *E. hormaechei* demonstrated the extensive diversity of this species complex, both at the phylogenetic and genomic levels. This research highlights *E. hormaechei* as a formidable pathogen with significant implications for human health. Its capacity for genotypic, and concomitantly phenotypic, flexibility, its potential for environmental persistence, as well as its role as multidrug resistant pathogen that may drive the spread of antibiotic resistance place emphasis on the need for continuous and thorough surveillance and the development of robust infection control measures to mitigate the impact of *E. hormaechei* on human health.

## Methods

### Genome sequences

The genomes of 3,499 *E. hormaechei* complex strains were obtained from the NCBI database [8]. These include a number of taxa previously ascribed to other *Enterobacter* species, including *E. cancerogenus* (1 strain), *E. cloacae* (93), *E. roggenkampii* (1), as well as 77 strains not assigned to the species level and six strains ascribed to other genera (*Escherichia, Klebsiella, Pedobacter* and *Pluralibacter*). Initial taxonomic delineation was determined using GTDB-tk v. 2.4.0 [57]. Genome completeness and contamination were assessed using BUSCO v. 5.2.2 [58] with the enterobacterales_odb10 lineage dataset (440 BUSCOs) and CheckM v. 2 [59], where only genomes with > 97% and < 3% contamination were retained for analysis. Following taxonomic and genome completeness curation, 3,387 *E. hormaechei* complex strains were retained (Supplementary Material 1 Table S1). After initial clustering, 50 genomes per clade were selected from each clade for further analysis, with the most complete genomes (least number of contigs – maximum 127 contigs) selected, along with the relevant type strains for each species/subspecies.

### Phylogenomic analyses

The 3,387 *E. hormaechei* complex strains were first clustered by calculating pair-wise average nucleotide identity values using FastANI v. 1.34 [10]. The resultant matrix was converted into a distance matrix (1—% ANI/100) and used to construct a neighbour-joining (NJ) distance tree using the *neighbor.exe* function in Phylip v. 3.698 [11]. The genomes of 50 taxa (and *E. quasihormaechei* WCHEQ120003^T^ as outgroup) from each species/species clade were subjected to the Genome-to-Genome Distance Calculator v. 3.0 server [15] and JSpecies v. 1.2.1 [16] to calculate dDDH and TETRA values, respectively, which were subsequently used to construct distance trees as described above. The representative genomes of each *E. hormaechei* complex species/subspecies were structurally and functionally annotated using Prokka v. 1.14.6 [60] and the protein datasets were used to identify orthologues using OrthoFinder v. 2.5.5 [61]. A total of 3,308 SCOs conserved among all comparator taxa were individually aligned using the M-Coffee implementation of T-Coffee v. 13.46.0.919e8cb6 [62]. The alignments were concatenated and poorly aligned blocks were removed using GBlocks v. 0.91b [13]. A maximum likelihood phylogeny was constructed on the curated alignment using IQ-Tree v. 2.3.6 [63] with an optimal evolutionary model as predicted with ModelFinder [14] and bootstrap approximation (*n* = 1,000 replicates) with UFBoot2 [64] to support the core genome phylogeny.

### Pan-genome analyses

The OrthoFinder output matrices were converted into presence/absence (1/0) matrices for each *E. hormaechei* complex species/subspecies and used to predict the pan- and core genome using the Bacterial Pan-Genome Analysis Pipeline (BPGA) v. 1.3 [65], followed by plotting and extrapolation using PanGP v. 1.0.1 [20]. Extrapolation of the pan- and core genomes, to determine their sizes if 100, 500 and 1,000 genomes would be added to the analysis, was performed by fitting the curves to Heap’s law [19]. The pan-genome was extrapolated with the formula *y* = *Ax*^*B*^ + *C*; where *y* is the pan-genome size, *x* the number of compared genomes and *A*, *B* and *C* are the curve fitting parameters. The core genome was extrapolated with the formula *y* = *Ae*^*Bx*^ + *C*; where *y* is the core genome size, *x* the number of compared genomes and *A*, *B* and *C* are the curve fitting parameters [19, 20]. The number of new genes when 100, 500 and 1,000 genomes would be added to the analysis was calculated using the formula *y* = *Ax*^*B*^; where *y* is the number of new genes, *x* the number of compared genomes and *A* and *B* are the curve fitting parameters [11, 18].

The core (proteins conserved among all taxa), accessory genome (proteins conserved among some but not all taxa) and singleton (proteins unique to a single taxon) pan-genome elements for each combination of species/subspecies were determined from the OrthoFinder matrices. For the inter-species comparison, 50 strains (16 subsp. *oharae,* 17 subsp. *steigerwaltii* and 17 subsp. *xiangfangensis*) were selected from the *E. xiangfangensis* (150 genomes) clade, along with the 50 *E. hoffmanni* and 50 *E. hormaechei* genomes, while 50 genomes each were selected for the inter-*E. xiangfangensis* subspecies comparisons. Representative proteins of each pan-genome element were extracted and functionally annotated using eggnog-mapper v. 2 [66] against the EggNOG v. 6.0 database [67]. Annotated proteins were clustered according to their Conserved Orthologous Group (COG) functions [21].

### Detection of mobile genetic elements, pathogenicity determinants and the resistome

Plasmids were detected by comparing the genome sequences against the Enterobacteriales dataset on the PlasmidFinder v. 2.1 server [22], while integrated bacteriophage elements were predicted using the PHASTEST server [23]. Integrative and Conjugative Elements (ICE) were identified by comparing the genomes against the ICEberg 3.0 database using ICEfinder 2.0 [25]. Transposable elements were detected using the Composite Transposon Finder (TnComp_finder) [26], where BLASTN v. 2.12.0 + [68] was used to compare the genomes (with the default parameters of 90% nucleotide identity and 95% query/subject coverage) against the default TnComp_finder transposon database.

The probability that the *E. hormaechei* complex taxa represent human pathogens was determined by evaluating the proteomes of the 250 comparator taxa against the PathogenFinder 1.1 server [28]. Pathogenicity determinants were subsequently identified in the proteomes of each strain by comparison against the Pathogen-Host Interaction (PHI) Database v. 5.0 [30], using BLASTP v. 2.12.0 + [68] using the parameters > 70% amino acid identity and > 70% query/subject coverage. To elucidate differences in the secretome, secretion systems were predicted using MacSyFinder v. 2 [34] with the TXSScan model [35].

To gain insight into the resistome of the *E. hormaechei* complex taxa, the proteomes were compared against the BacMet database v. 2.0 [46] using BLASTP v. 2.12.0 + [68] with the cut-off parameters the parameters > 70% amino acid identity and 70% query/subject coverage. Antibiotic resistance phenotypes were further predicted by comparing the genome sequences against the ResFinder v. 4.6 server [44].

## Supplementary Information

Supplementary Material 1.

## Data Availability

All data pertaining to this study are available in this publication and its supplementary data. The genome sequences utilised in the study are publicly available in the NCBI Genome Assembly database and the accession numbers for the genome sequences are indicated in Supplementary Material 1 (Tables S1 and S3).
